# Identifying Pelagic Habitat Hotspots of Neon Flying Squid in the Temperate Waters of the Central North Pacific

**DOI:** 10.1371/journal.pone.0142885

**Published:** 2015-11-16

**Authors:** Irene D. Alabia, Sei-Ichi Saitoh, Robinson Mugo, Hiromichi Igarashi, Yoichi Ishikawa, Norihisa Usui, Masafumi Kamachi, Toshiyuki Awaji, Masaki Seito

**Affiliations:** 1 Arctic Research Center, Hokkaido University, N21 W11 Kita-Ku, Sapporo, 001–0021, Japan; 2 Laboratory of Marine Environment and Resource Sensing, Graduate School of Fisheries Sciences, Hokkaido University, 3-1-1 Minato-cho, Hakodate, 041–8611, Hokkaido, Japan; 3 Regional Centre for Mapping of Resources for Development—SERVIR-Africa, Kasarani Road, P.O. Box 632–00618, Ruaraka, Nairobi, Kenya; 4 Data Research Center for Marine-Earth Sciences, Japan Agency for Marine Earth-Science and Technology, JAMSTEC, 3173–25 Showamachi, Kanazawa-ward, Yokohama City, Kanagawa, 236–0001, Japan; 5 Oceanographic Research Department, Meteorological Research Institute, 1–1 Nagamine, Tsukuba, 305–0052, Japan; 6 Graduate School of Science Division of Earth and Planetary Science, Kyoto University, Sakyo-ward, Kyoto, 606–8502, Japan; 7 Aomori Prefectural Industrial Technology Research Center, 4-11-6 Dainitonya-machi, Aomori-shi, Aomori, 030–0113, Japan; Institute of Marine Research, NORWAY

## Abstract

We identified the pelagic habitat hotspots of the neon flying squid (*Ommastrephes bartramii*) in the central North Pacific from May to July and characterized the spatial patterns of squid aggregations in relation to oceanographic features such as mesoscale oceanic eddies and the Transition Zone Chlorophyll-*a* Front (TZCF). The data used for the habitat model construction and analyses were squid fishery information, remotely-sensed and numerical model-derived environmental data from May to July 1999–2010. Squid habitat hotspots were deduced from the monthly Maximum Entropy (MaxEnt) models and were identified as regions of persistent high suitable habitat across the 12-year period. The distribution of predicted squid habitat hotspots in central North Pacific revealed interesting spatial and temporal patterns likely linked with the presence and dynamics of oceanographic features in squid’s putative foraging grounds from late spring to summer. From May to June, the inferred patches of squid habitat hotspots developed within the Kuroshio-Oyashio transition zone (KOTZ; 37–40°N) and further expanded north towards the subarctic frontal zone (SAFZ; 40–44°N) in July. The squid habitat hotspots within the KOTZ and areas west of the dateline (160°W-180°) were likely influenced and associated with the highly dynamic and transient oceanic eddies and could possibly account for lower squid suitable habitat persistence obtained from these regions. However, predicted squid habitat hotspots located in regions east of the dateline (180°-160°W) from June to July, showed predominantly higher squid habitat persistence presumably due to their proximity to the mean position of the seasonally-shifting TZCF and consequent utilization of the highly productive waters of the SAFZ.

## Introduction

The complex environment of pelagic oceans provides a broad range of habitats exploited by marine organisms at different stages of their life history. Distinct spatial environmental features are often recognized and used by marine organisms for different biological and physiological functions to ensure their survival. For instance, pelagic species are typically associated with certain environmental factors and mesoscale oceanographic features such as meandering front and eddies [1, 2, 3]. These oceanographic features thus in turn, are relevant to the formation of “hotspots”, which are defined as regions of intense biological activity and persistent species aggregations driven by enhanced trophic interactions and foraging conditions and are mostly targeted by the commercial and artisanal fishery [4, 5]. Hence, the oceanic hotspots constitute important biological features of high ecological and economic importance. With the heightened exposure of marine ecosystems to various anthropogenic and climate perturbations, identifying and characterizing the marine hotspots are central for setting up conservation priorities and evaluating resource management strategies [6, 7, 8].

Neon flying squid (*Ommastrephes bartramii*) is a large cephalopod widely distributed in the subtropical and temperate oceans [9]. It is one of the internationally (i.e. Japan, China, South Korea and Taiwan) exploited fisheries of high commercial value in the North Pacific and together with other squid species, it accounts for approximately 5% of the total landings in the region [10]. The commercial fishery for this species started when the catches of Japanese common squid (*Todarodes pacificus*) dramatically decreased in the 1970s [11] and commercial harvesting of the neon flying squid resources in the North Pacific continued since then. On the ecological perspective, *O*. *bartramii* further assumes important ecological roles in the pelagic ecosystem owing to its mid-trophic position in the oceanic food webs [12]. Previous studies on the feeding habits and diet of neon flying squid revealed that it generally preys on crustaceans, small pelagic fishes and cephalopods [13, 14]. It is also an important prey species for the larger predators [15, 16] and is thereby thought to create important connections between animals of tertiary trophic level and top predators.

Moreover, in the North Pacific, *O*. *bartramii* population is comprised of two seasonal spawning cohorts (winter-spring and autumn) with life histories extremely dependent on the variability of spatial and temporal features of the oceanographic environment [17, 18, 19, 20]. For instance, the squids’ annual roundtrip north-south migration for feeding and spawning is linked with the northern warm-water branches and the warm-core rings of the Kuroshio Current [21, 22]. The differences in the body size between the two cohorts, with the autumn spawning cohorts generally larger than the winter-spring cohorts, are further traced from variations in the oceanic environments off their spawning/nursery and northward migration habitats [23, 24]. Specifically, the spatial and temporal mismatches on the utilization of frontal feature such as the Transition Zone Chlorophyll-a Front (TZCF) [25] between the two cohorts during the early life stages, accounted for their growth differences.

Species-environment studies using predictive models are based on the hypothesis that the species optimal habitats are shaped by biological and environmental controls [26, 27, 28]. In the marine ecosystem, oceanographic variables are frequently used as proxy indicators to examine the effects of biophysical processes to species distribution and abundance [29, 30, 31, 32]. In the present work, we selected the most parsimonious set of environmental variables that significantly influenced the monthly potential habitat of neon flying squid in the central North Pacific from a larger pool of biologically-important and available environmental data using the Maximum entropy (MaxEnt) [33] models. From spatial predictions of squid suitable habitat, we further investigated the stability of these regions and developed a simple measure of habitat persistence to identify potential squid habitat hotspots in the central North Pacific. The spatial and temporal dynamics of the observed squid habitat hotspots were subsequently described in relation to the presence of oceanic eddies and mean geographical positions of the TZCF.

## Materials and Methods

### Squid fishery information

**Fig 1:**
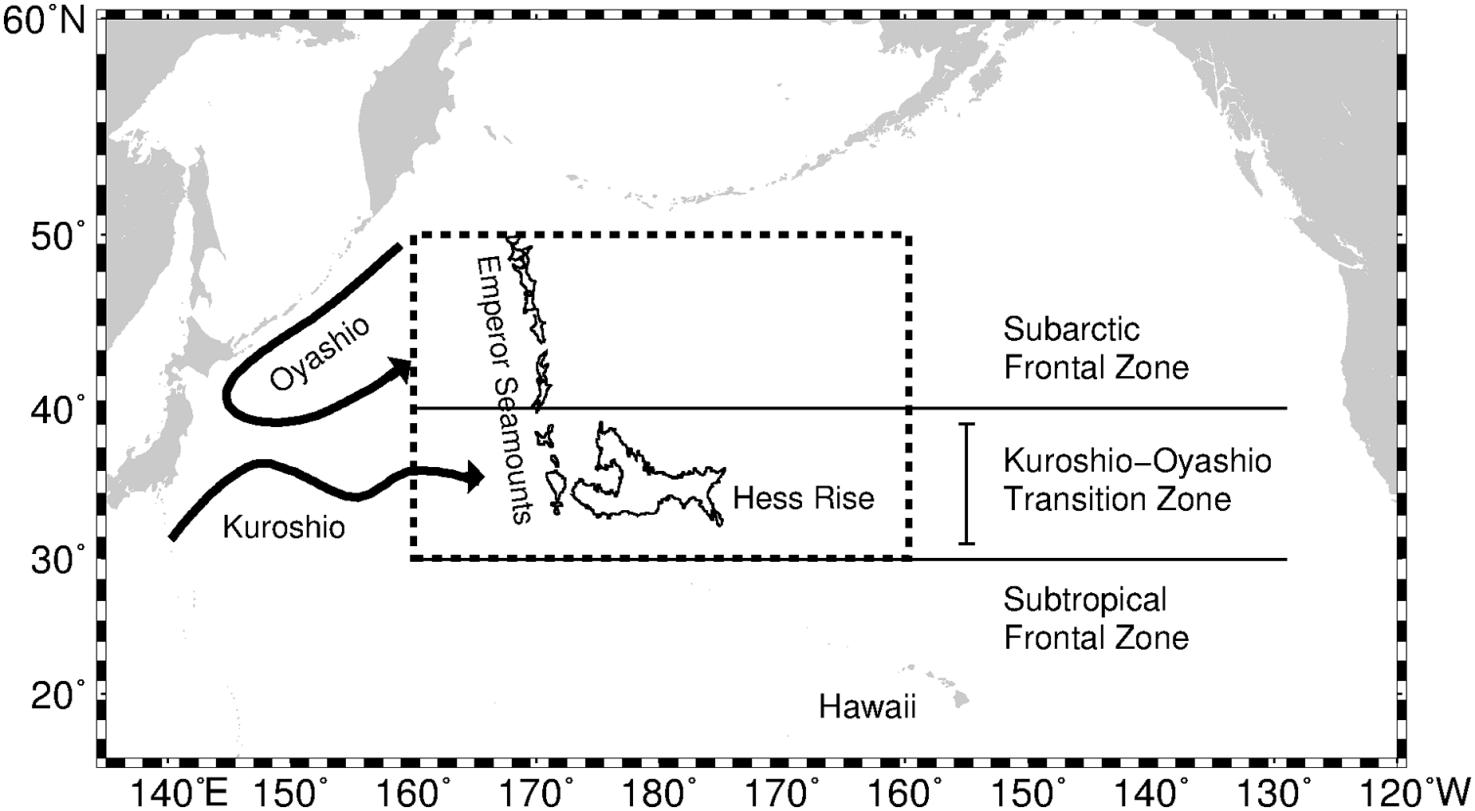
Map of the North Pacific showing the major oceanographic and topographic features. The broken lines correspond to the Japanese squid fishing region in the central North Pacific.

Squid fishing locations were provided by Aomori Prefectural Industrial Technology Research Center (APITRC) during the squid fishing periods from May to July, 1999–2010, covering the Japanese jigging area in the central North Pacific (170°E-160°W; 30–50°N; Fig 1). The dataset contained the point data information on the daily commercial jigging vessel fishing locations (Longitude, Latitude) and fishing date (Month, Day, Year), where all the squid occurrence data used for habitat model analyses have positive (non-zero) squid catches (S1 Fig). These were subsequently pooled into the respective monthly databases for an effort-based habitat model development.

### Environmental variables

The environmental (dynamic and static) data used for the analyses were obtained from satellite measurements and numerical model products (Table 1). The remotely-sensed variables were sea surface temperature (SST), sea surface height (SSH), geostrophic velocity components (u, v), eddy kinetic energy (EKE), net primary productivity (NPP) and chlorophyll-*a* (Chl-*a*) while the numerical model-derived oceanographic variables were sea surface salinity (SSS) and mixed layer depth (MLD). The satellite data were downloaded from the National Oceanic and Atmospheric Administration (NOAA) coastwatch server (coastwatch.pfeg.noaa.gov/erddap/) except for NPP, which were obtained from Oregon State University (OSU) ocean productivity website (science.oregonstate.edu/ocean.productivity/) while the EKE was computed from the altimetry-derived geostrophic velocity components (u, v) [34]. The numerical model products were derived from 3-dimensional multi-variate ocean variational estimation (MOVE) system for Western North Pacific (MOVE-WNP), developed by the Japan’s Meteorological Research Institute (MRI) [35]. The static environmental variable examined was the bathymetric gradient (∇depth), computed using the ETOPO1 bathymetry data [36] derived from global relief model of Earth's surface, provided by the NOAA National Centers for Environmental Information (NCEI) (ngdc.noaa.gov/mgg/global/). The bathymetric gradient was derived using the slope function of SDMTools package [37]. Due to the differences in spatial and temporal resolutions, all environmental data were resampled into the coarsest available spatial footprint (25 km) and sampling interval (monthly) prior to habitat model construction.

**Table 1:** Summary of environmental parameters used for developing habitat models for neon flying squid in the central North Pacific.

Environmental Variables	Abbreviation	Sampling Interval	Spatial Footprint	Primary Source
Sea surface temperature	SST	Daily	25 km	AVHRR
Sea surface salinity	SSS	5-day mean	10 km	MOVE-MRI
Chlorophyll-*a*	Chl-*a*	Monthly	9 km	SeaWIFS
Net primary productivity	NPP	Monthly	9 km	SeaWIFS
Sea surface height	SSH	Daily	25 km	AVISO
Zonal geostrophic velocity	geostrophic_u	Daily	25 km	AVISO
Meridional geostrophic velocity	geostrophic_v	Daily	25 km	AVISO
Eddy kinetic energy	EKE	Daily	25 km	AVISO
Mixed layer depth	MLD	5-day mean	10 km	MOVE-MRI
Bathymetric gradient	∇dep	-	1-arc minute	ETOPO1

### Habitat model development

Fishing effort and catch per unit effort (CPUE) are generally considered as reliable proxies of species presence and abundance, respectively and have been increasingly used for developing habitat models [38, 39, 40]. However, for the neon flying squid, effort-based habitat models performed better than CPUE-based models in predicting the potential squid habitat as the latter tended to overestimate the ranges of the optimal squid habitats and underestimate the monthly variability in spatial distributions of the optimal habitats [20, 41]. Moreover, earlier studies on other fisheries have also shown that the fishing effort is a better index of resource distribution relative to CPUE in a system where potential competition for space on the best fishing grounds occurs [42, 43, 44]. Therefore, in this study we utilized the squid presence data with positive squid catches in relation to the parsimonious sets of environmental data, to identify the potential squid habitat in the central North Pacific. The habitat models were constructed within the information theoretic approach primarily based on maximum entropy (MaxEnt) algorithm [33]. MaxEnt is a model approach that determines the probability of suitable habitat for a species at each pixel within the geographic domain, by combining both environmental layers and species occurrence dataset [33]. MaxEnt model has been successfully used in a broad range of terrestrial [45, 46] and marine [47, 48] applications. In this paper, we used MaxEnt to identify and characterize the environmental parameters (both dynamic and static) that better describe the habitat of squids and consequently create the spatial predictions of potential squid habitat in the central North Pacific. The MaxEnt models were separately ran using the MaxEnt software, version 3.3.3k (cs.princeton.edu/~schapire/maxent/) for May, June and July from the monthly-pooled squid occurrences from 1999 to 2010 and initial suite of the environmental and topographic factors that could potentially influenced the squid habitat. The exploratory monthly MaxEnt habitat models were initially developed using the sample with data (SWD) (www.cs.princeton.edu/~schapire/maxent/tutorial/tutorial.doc), which contained the spatially-matched squid occurrences and the corresponding values of the 10 environmental layers at each presence point (Table 1). The background SWD file was subsequently created by pooling the randomly-selected pseudo-absences (500 points) for each of the environmental variable. The monthly squid presence data were then split into model training (70%) and testing (30%) data to evaluate the models’ predictive performance and select the most important environmental variables to squid habitat, using Area under the curve (AUC) of the Receiver Operating Characteristic (ROC) [49] and percent variable contribution, respectively. Following the environmental layer selection from preliminary analyses, the 3 most influential factors for each monthly models based on the percent variable contribution, were then used to construct the final MaxEnt models for predicting the potential squid habitats from May to July, 1999–2010. The response curves generated from each models were then examined to deduce the environmental ranges characterizing the potential squid habitat in the Central North Pacific.

### Spatial patterns of habitat predictions

The monthly habitat predictions derived from the MaxEnt models were expressed in a habitat probability metric, hereafter referred to as habitat suitability index (HSI), with values ranging from 0 (not suitable) to 1 (most suitable). To examine the monthly spatial squid habitat patterns, HSI predictions were averaged monthly and the pixel-wise standard deviations (SD) were then computed from 1999 to 2010. Pixel-wise SDs provide a measure of uncertainty for the monthly HSI predictions across the 12-year period [50]. The regions with high and low HSI SDs are interpreted as the areas with variable and stable squid potential habitats, respectively.

### Spatiotemporal overlap of squid potential habitat

The spatial and temporal overlap of predicted squid habitats from May to July 1999–2010 were designed to identify the potential squid habitat hotspots in the Central North Pacific. Using this measure, potential habitat hotspots for squids are defined by high temporal overlap, that is, the number of years that a pixel was predicted as a suitable habitat based on a given threshold. For this purpose, HSI threshold used to classify the monthly squid habitat predictions into 0 (not suitable) and 1 (suitable) were arbitrarily represented by the 25^th^ percentile of HSI distribution extracted from the actual squid fishing locations. The monthly binary maps were then summed up and divided by the entire duration of the study (12 years) to determine the relative pixel-wise potential habitat overlap over time, thereby, creating a simple habitat persistence index, hereafter referred to as the HPI, with values ranging from 0 to 1. Using this index, squid habitat hotspots correspond to the regions with high HPI and its monthly spatial distributions in central North Pacific were subsequently mapped. All the mapping routines were implemented using generic mapping tools (GMT) version 4.5.12.

### Oceanographic features and squid habitat hotspots

After identifying the regions of potential habitat hotspots for squid in the Central North Pacific, we briefly explored the relevance of oceanographic features such as the seasonally-migrating transition zone chlorophyll-*a* front (TCZF) and oceanic eddies to the squid potential habitat hotspots. TZCF is one of the important frontal features in the North Pacific that affects foraging condition and migration habitat for many pelagic species [51, 52, 53]. Inter-annual variation in the TZCF positions also resulted to the changes in neon flying squid abundance [54]. Here, the climatological means of SeaWIFS chlorophyll-*a* data for May, June and July 1999–2010 were computed and monthly climatological positions of the TZCF, defined by 0.2 mg·m^3^ [25], were subsequently mapped. The other relevant oceanographic feature examined are eddies and were also documented to significantly influence fisheries [55, 56]. Oceanic eddies within 160°E-160°W and 30°-50°N were detected from the daily altimetry-derived SSH data using the eddy detection routines [57] implemented in MATLAB R2014a. The detected eddies with lifespan ≥ 30 days were then classified into either warm core (anticyclonic) and cold core (cyclonic) eddies and the number of eddy centers within a 25 km pixel were summed up for each month from 1999 to 2010. The outermost spatial boundaries of the potential squid habitat hotspots (HPI = 0.25) were subsequently extracted and their contours were overlain on the spatial maps of chlorophyll-*a* with the TZCF position and eddy counts.

## Results

### Spatial patterns of squid aggregations and environmental variables

**Fig 2:**
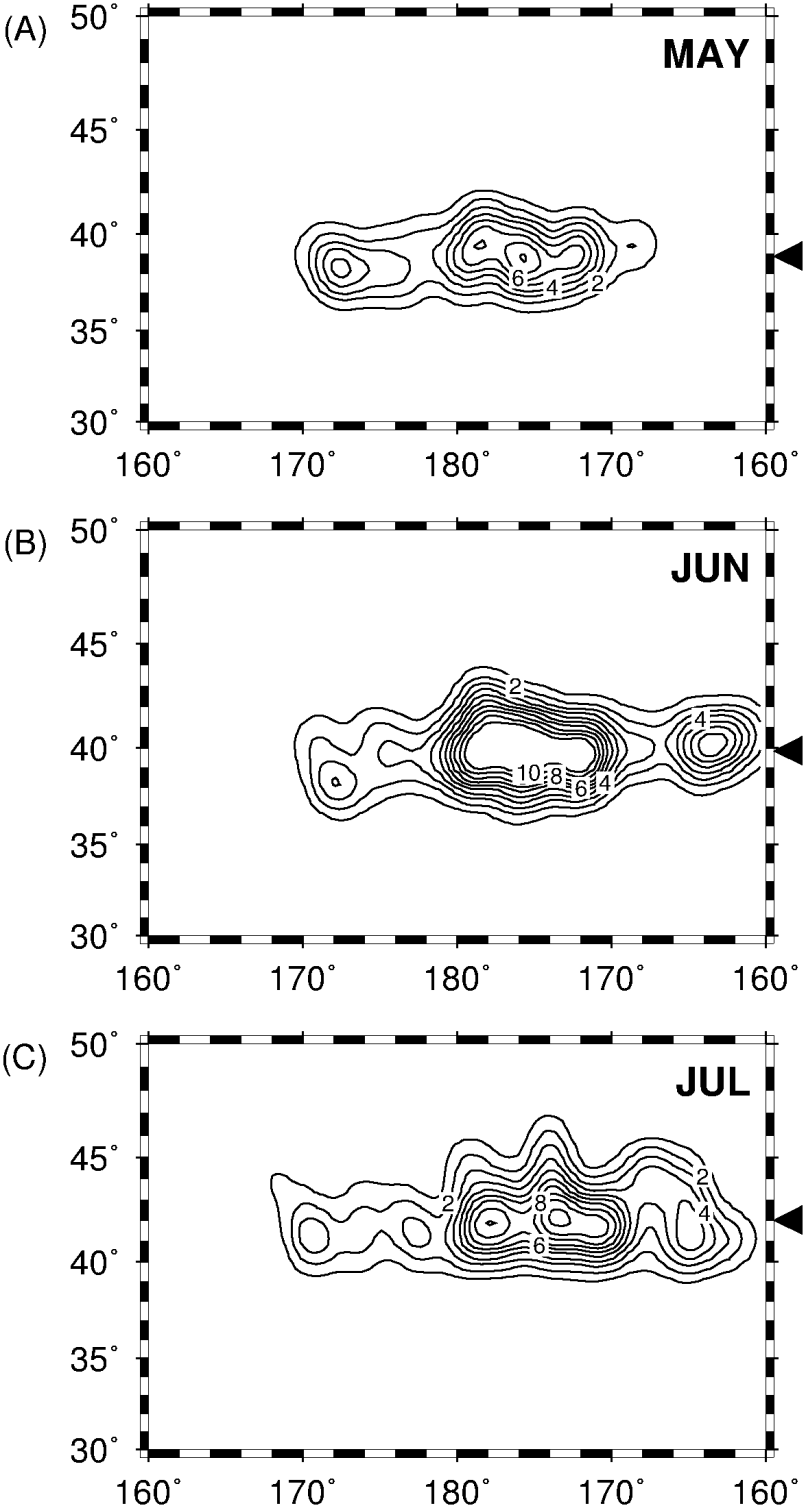
Kernel density distribution of squid fishing locations (representing squid fishing effort) for (A) May, (B) June and (C) July 1999–2011. Japanese summer squid fishing activities are mainly concentrated west of 180°. Latitudinal distribution centroids are shown by the triangles on the right.

Fig 2A–2C shows the kernel density distributions of monthly-pooled squid fishing locations, corresponding to squid fishing effort from May to July, 1999–2010. In May (Fig 2A), squid fishing effort is primarily concentrated across 170°E-170°W, where the highest density occurs west of the dateline (180°-170°W). In June (Fig 2B), the squid fishing effort extended further west, generating a second density peak between 170°E and 160°W with the latitudinal distribution centroid (39.84°N) slightly shifting to the north. In July (Fig 2C), the squid fishing activities maintained their zonal extent and the fishing vessel density peaked across 180°-170°W, while shifting the latitudinal distribution centroid (42.03°N) further north.

**Fig 3:**
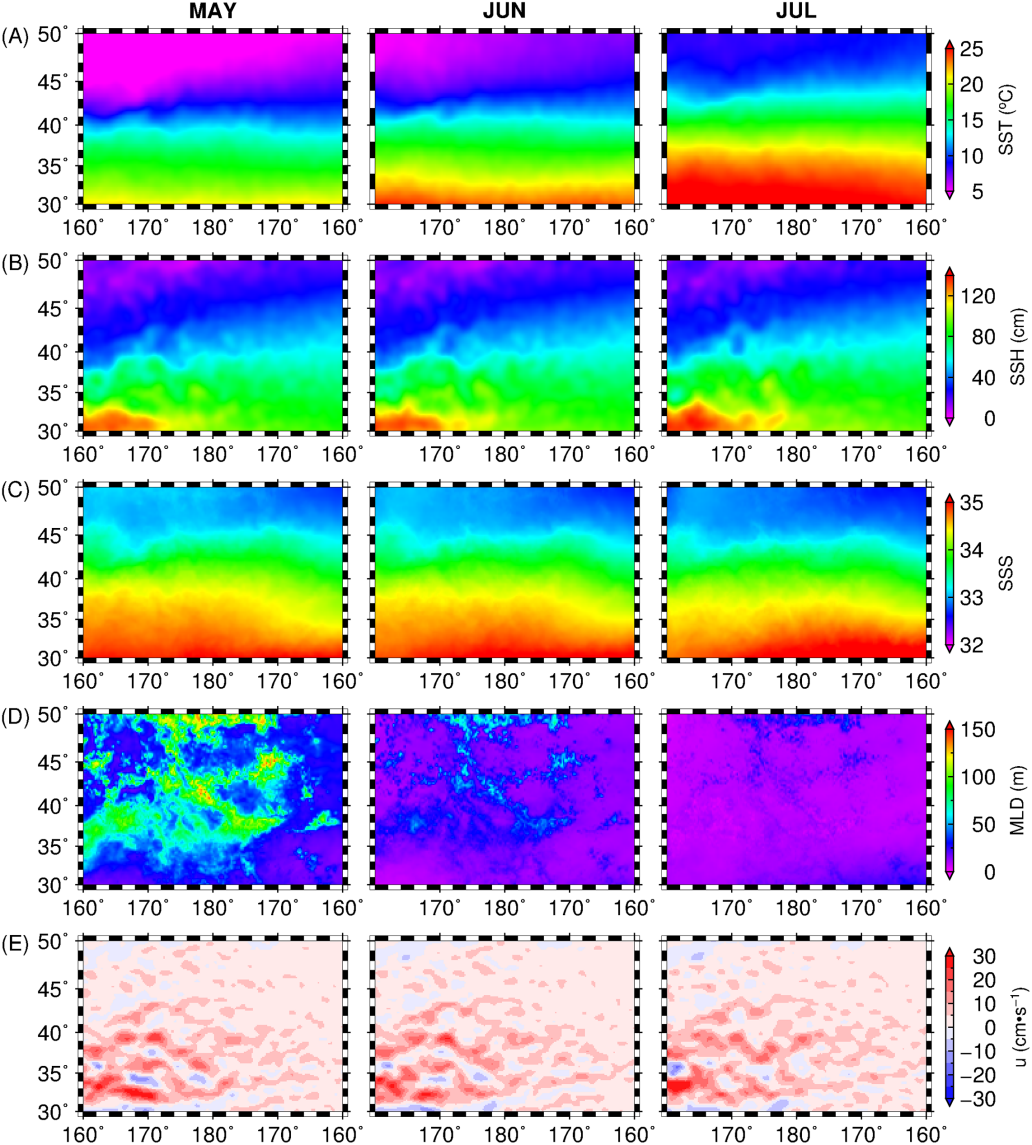
Spatial maps of the environmental variables used for the final monthly MaxEnt models represented by (A) SST, (B) SSH, (C) SSS, (D) MLD and (D) u averaged from 1999 to 2010 across the study area.

Fig 3A–3E shows the spatial maps of the 12-year long climatological mean for environmental variables used in the final monthly MaxEnt models from May to July (Table 2). Monthly SST (Fig 3A) spatial patterns showed warming signals that are likely driven by seasonal changes in insolation while the spatial patterns of SSH (Fig 3B) showed zonal expansion of the warm Kuroshio Current extension across 30–35°N from May to July. The spatial patterns of SSS (Fig 3C) showed the strongest freshening signals between 160°W-180° and 40–50°N in May, which gradually weaken from June to July. This could potentially mirror the seasonal variability in the strength of Oyashio intrusion and transport, generally exhibiting maximum in winter-spring [58]. MLD spatial maps (Fig 3D) further showed shoaling patterns from May to July with deepest MLD in the subarctic region (40–50°N). This response could be driven by seasonal relaxation of wind patterns in the North Pacific [59]. Finally, the spatial patterns of the zonal geostrophic current component (u) from May to July, also revealed predominantly eastward flow, with highest magnitude observed along the path of the Kuroshio Current extension between 160°E-180° and 30–40°N.

**Table 2:** The relative model contribution of the environmental parameters used for initial habitat model construction for May, June and July, 1999–2010.

Environmental variables	Percent Contribution		
	May	June	July
SST	**61.3**	**70.6**	**58.2**
SSS	2.7	0.3	**16.7**
SSH	**24.0**	**24.9**	**19.8**
EKE	0.6	0.2	0.5
geostrophic_u	0.1	**2.1**	0
geostrophic_v	0.0	0.2	1
MLD	**9.3**	0.8	2.7
NPP	1.3	0.2	0.3
Chl-*a*	0.1	0.7	0.2
∇depth	0.6	0.1	0.4

Bold values correspond to the contributions of the 3 most important variables for final model development.

### MaxEnt model environmental variable selection

**Fig 4:**
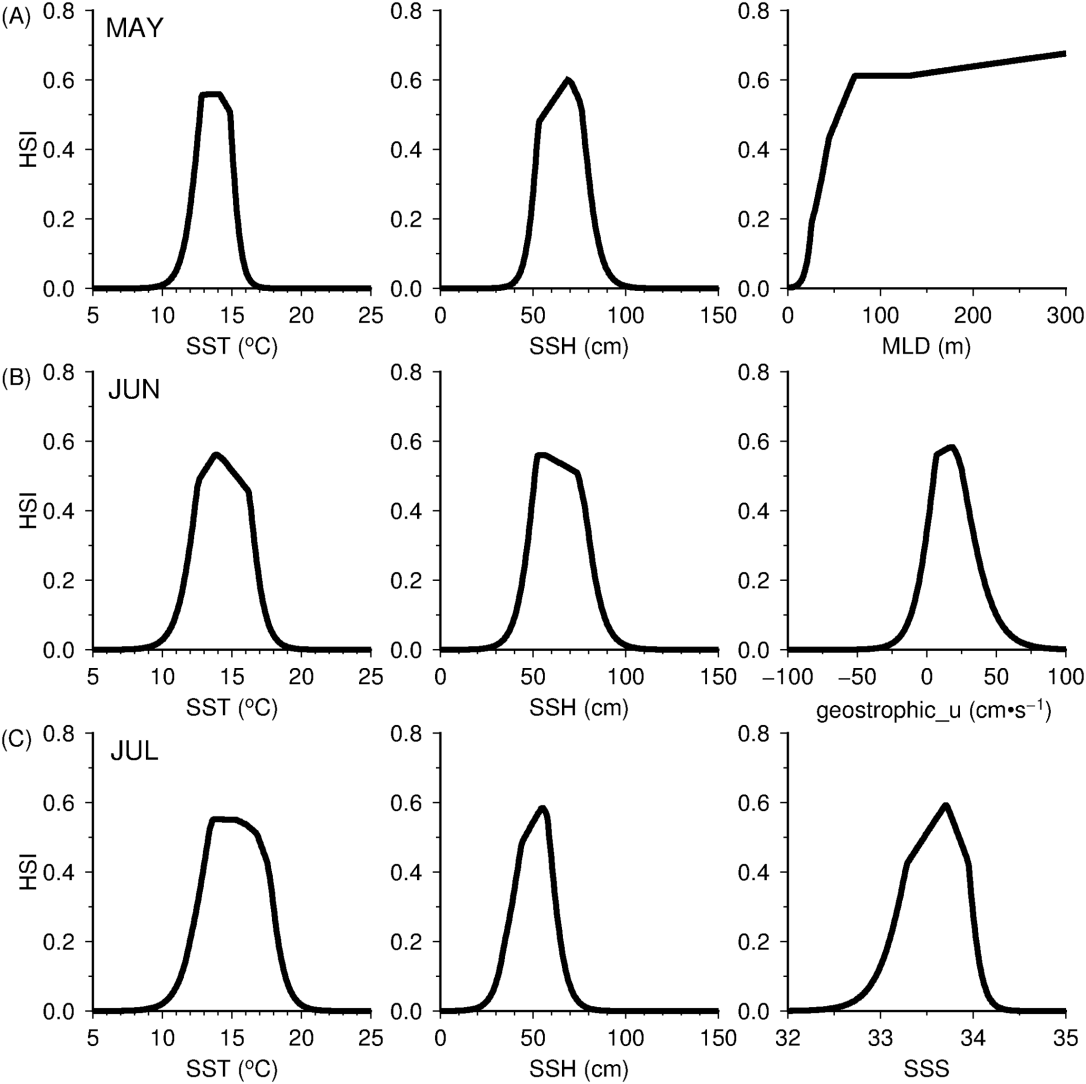
Response curves for the final environmental variables used for (A) May, (B) June and (C) July MaxEnt models.

Table 2 shows the relative variable contribution of the initial pool of environmental predictors used to develop the monthly MaxEnt habitat models. These preliminary results revealed that SST and SSH showed constantly high variable importance (78–96%) to squid habitats in May, June and July. However, the environmental variables that ranked after SST and SSH across the monthly models varied on their relative model contributions. In May, for instance, MLD (9.1%) ranked as the third important variable while in June and July, it was geostrophic_u (2.1%) and SSS (19.8%), respectively. The final monthly models corresponded to the most parsimonious habitat models, using the top 3 environmental factors (accounting for 95–98% of the predicted spatial squid habitat patterns) that significantly influenced the squid potential habitat in central Pacific. Fig 4A–4C showed the derived response curves from the final 3-parameter monthly models. In May (Fig 4A), the squid potential habitat was defined by the SST, SSH and MLD ranges from 10–16°C, 40–90 cm and 0–300 m, respectively. The elevated MLDs showed positive effect on the squid habitat during this period. In June (Fig 4B), SST and SSH covered wider ranges, with SSH extending into colder regions. During this month, the squid potential habitat was also defined by relatively strong eastward flow (-25-75 cm·s^-1^). In July (Fig 4C), squid potential habitat was characterized by SST, SSH and SSS with ranges between 10–20°C, 20–80 cm and 32.5–34.1, respectively.

### Spatial patterns of squid habitat and prediction uncertainty

**Fig 5:**
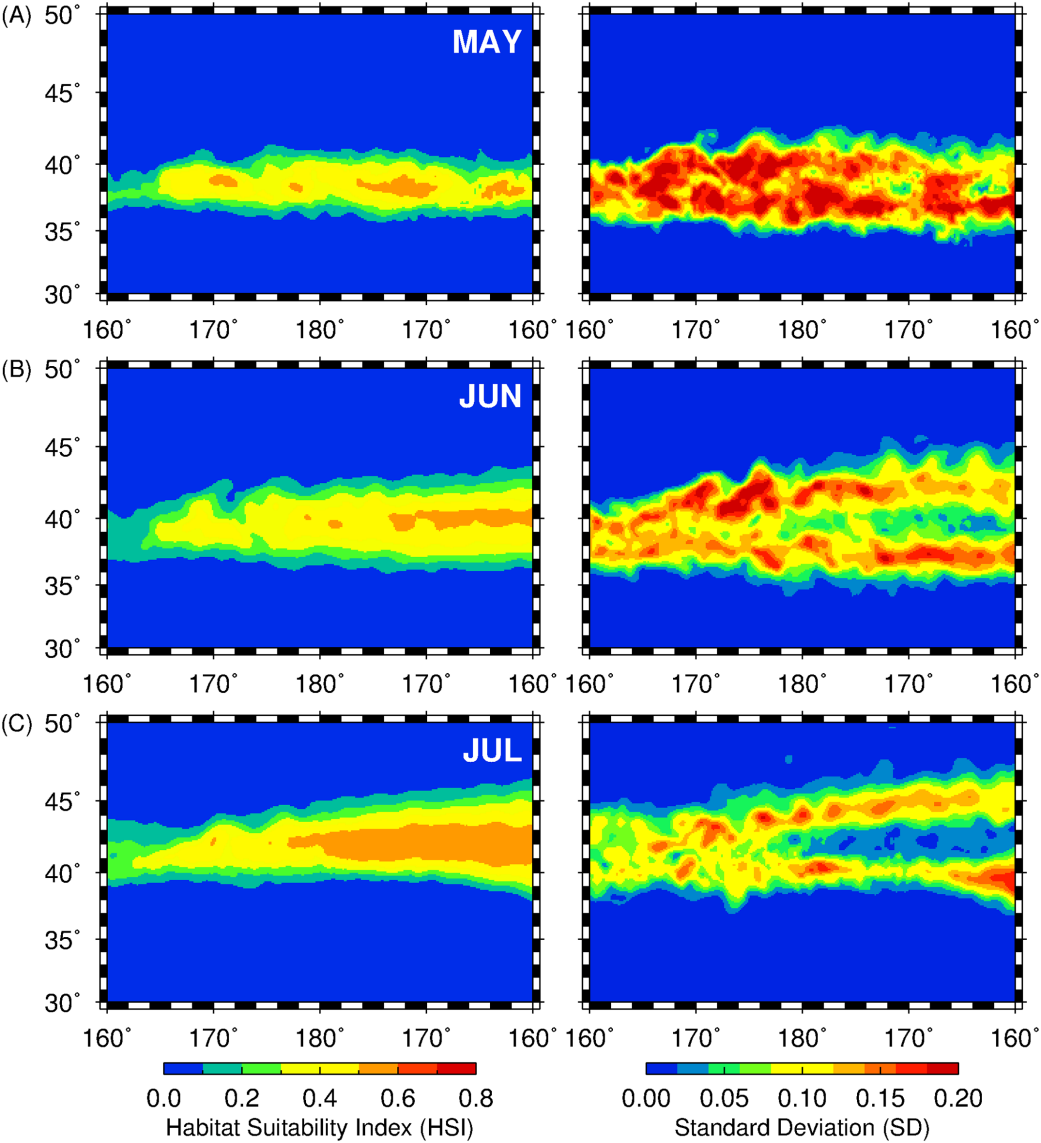
Spatial HSI (left panel) and standard deviation maps (right panel) averaged from 1999 to 2011 for (A) May, (B) June and (C) July.

Fig 5A–5C showed the monthly-averaged predicted squid habitats with their corresponding uncertainty maps (standard deviation) from 1999 to 2010. The spatial patterns between monthly predictions exhibit differences such that squid potential habitat increased in spatial extent and magnitude, with decreased in prediction uncertainties from May to July. In May (Fig 5A, left panel), squid potential habitats were found between 36–41°N with interspersed patches of high suitable regions (HSI ≥ 0.5) that are further defined by high prediction uncertainty (high SD; Fig 5A, right panel). Prediction uncertainty levels show as to whether the predicted habitat is stable or not over the 12-year period and hence, highlight the transient nature of squid potential habitats in May. In June (Fig 5B, left panel), squid potential habitat formed between 37–44°N, with high suitable areas west of the dateline, further defined with low corresponding SD (right panel). As the summer season progresses, the squid potential habitat in July (Fig 5C, left panel) shifted north between 38–47°N, with high suitable areas expanding from 180° to 160°W. East of 180°, however, patches of high suitable areas were less evident for June and July and spatial patterns of associated habitat uncertainty in these regions were correspondingly higher relative to the western regions (180°-160°W).

### Spatial patterns of temporal overlap of squid habitat

**Fig 6:**
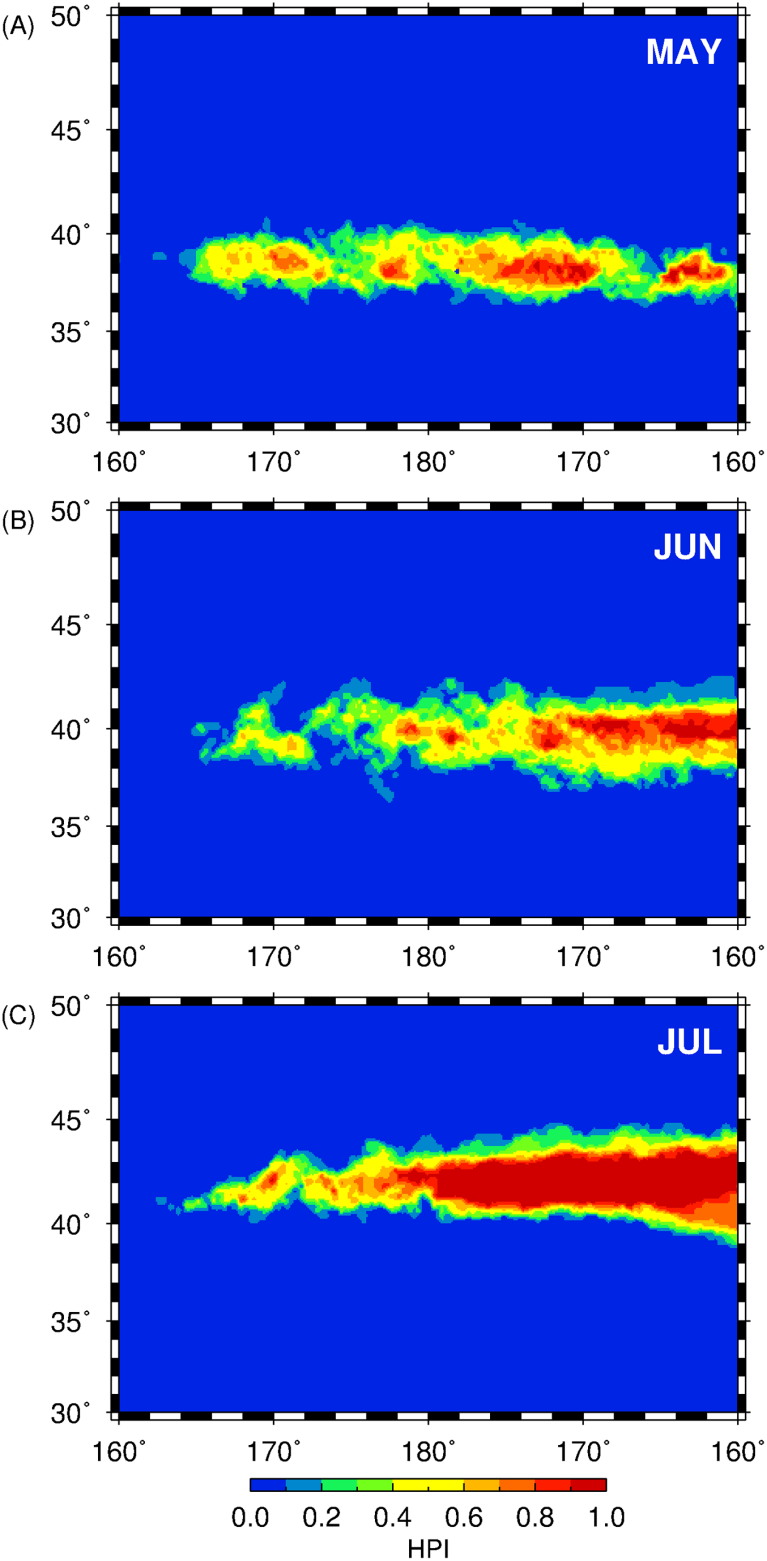
Spatial maps of HPI for (A) May, (B) June and (C) July 1999–2011. HSI thresholds used for binary classification were derived using the 25^th^ percentile of the HSI extracted at the actual fishing locations for May, June and July, 1999–2010.

The spatial distributions of the temporal overlap of squid habitat from May to July, 1999–2010 are shown in Fig 6A–6C and HSI thresholds used for the computation are shown in Table 3. In May, the squid potential habitat hotspots were generally patchy, with distribution between 170°E-160°W. The biggest squid hotspot patch for this period also developed between 176°W-169°W and 37–39°N. In June, squid habitat hotspots were concentrated between 174°W-160°W with a slight meridional shift between 39–40°N. In July, squid habitat hotspots further expanded north (40–44°N) covering the widest spatial extent relative to the other months (176°E-160°W). The regions west of the dateline (160°E-180°), however, showed low HPI across all the months, with the widest spatial extent during May, when squid habitats were mainly located in the south (37–40°N) relative to other months.

**Table 3:** Interquartile range of HSI distribution from the monthly-pooled actual squid fishing locations from May to July, 1999–2010.

MaxEnt models	Habitat Suitability Index (HSI) Quantiles				
	0%	25%	50%	75%	100%
May	0.04	0.48	0.54	0.59	0.65
June	0.01	0.47	0.53	0.56	0.65
July	0.06	0.47	0.54	0.57	0.62

The monthly 25^th^ HSI percentiles were used as thresholds for the binary classification of monthly squid habitat predictions to derive temporal habitat overlap as an index of habitat persistence (HPI).

### Squid habitat hotspots in relation to oceanographic features

**Fig 7:**
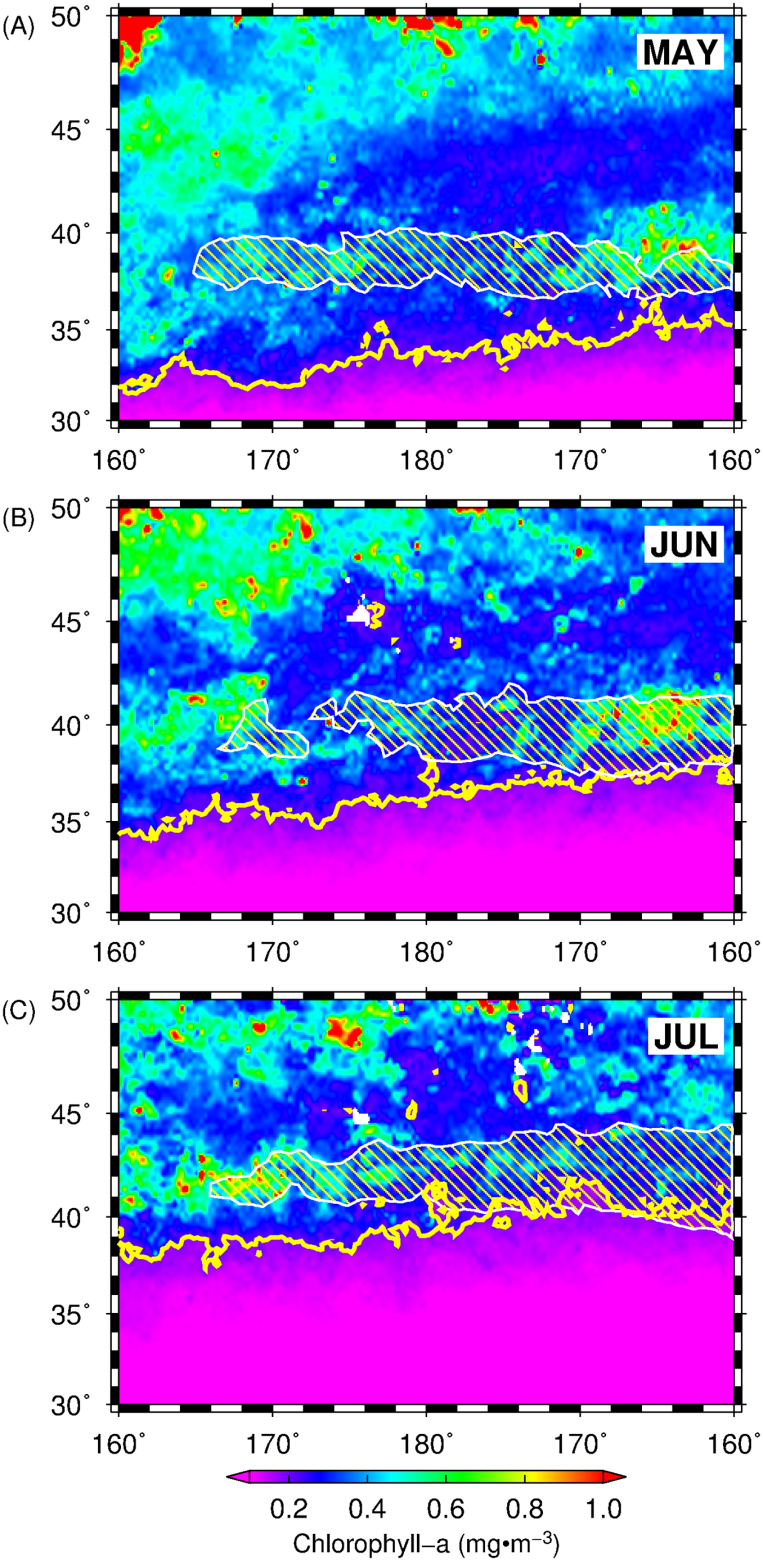
Maps of climatological averages of chlorophyll-*a* and location of the TZCF (0.2 mg/m^2^ of chlorophyll-*a*; yellow solid lines) from (A) May, (B) June and (C) July, 1999 to 2010. White polygon corresponds to the outermost boundaries of squid habitat with temporal overlap of 3 years (HPI = 0.25).

The outermost boundaries of the monthly squid habitat hotspots (HPI = 0.25, corresponding to a temporal habitat overlap of 3 years) overlain on the climatological averages of chlorophyll-*a* and contours representing the monthly position of the TZCF are shown in Fig 7A–7C. Despite of the small variable contribution of chlorophyll-*a* to the initial MaxEnt models for predicting squid habitat, the identified squid habitat hotspots from May to July, 1999–2010 corresponded well with the regions of high primary productivity and that the southern boundary of the squid habitat hotspots east of the dateline (180°-160°W) were found close to the climatological mean position of the TZCF especially during the months of June and July. These results suggest that the TZCF could likely influence the foraging conditions of the neon flying squid in summer and consequently impact the persistence of its suitable habitat.

**Fig 8:**
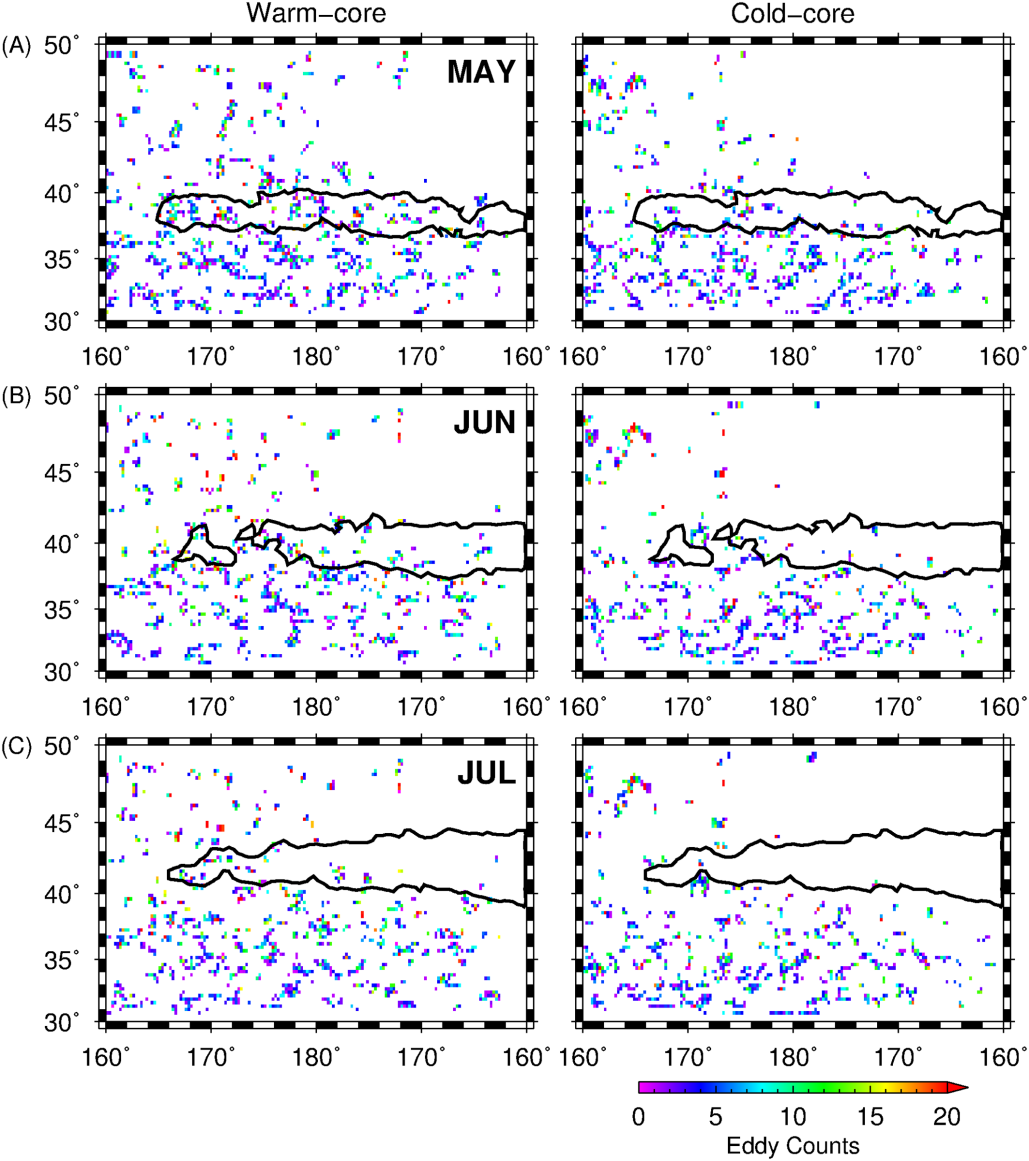
Spatial map of pixel-wise counts of warm-core (left panel) and cold-core (right panel) eddy for (A) May, (B) June and (C) July, 1999 to 2010. Black polygon corresponds to the outermost boundaries of squid habitat with temporal overlap of 3 years (HPI = 0.25).

Further analyses of the spatial and temporal distributions of oceanic eddies in the central North Pacific revealed the eddy-rich zones dominated by warm and cold-core eddies, between (1) 160°E-160°W and 30–40°N and (2) 160°E-180° and 30–50°N (Fig 8A–8C) which also follow the zonal gradients of Kuroshio and Oyashio western boundary flows. In May, the squid habitat hotspots distribution were mainly located within the first region while from June and July the possible associations of squid habitat hotspots to the eddy features east of 180° is likely reduced as the squid habitat pattern shifted northeast. However, in regions west of the dateline (160°E-180°) corresponding to the zonal extent of Kuroshio extension, the presence of warm- and cold-core eddies could still potentially influence the dynamics of the squid habitat hotspots.

## Discussion

We have explored and identified the pelagic squid potential habitat hotspots in the central North Pacific by combining available environmental variables and fishery information as input to the MaxEnt habitat models. The spatial and temporal patterns of these biological features were also determined based on the 2 criteria: (1) high habitat suitability and (2) persistence of the highly suitable habitat for neon flying squid over time. Within the 12-year period, the squid potential habitat hotspots showed differences in spatial patterns from May to July and we proposed that these variations are likely due to the dynamics of oceanographic features to which, the squids are associated with, during the course of their northward feeding migration off the central North Pacific from late spring to summer. For instance, in May when the squids primarily feed within the Kuroshio-Oyashio transition zone (KOTZ), the squid habitat hotspots were comprised of small patches of persistent suitable habitats (Figs 5A and 6A) relative to June and July. The KOTZ (140°E-160°W, 35°-40°N) is a highly dynamic oceanographic region where both warm and cold-core eddies are distributed [60, 61]. The distribution of these eddies along this zone resulted to the local enhancement of primary productivity that provide favorable foraging opportunities for pelagic species [39, 62]. In this study, the association of squid to the transient and dynamic oceanic eddies in the KOTZ (160°E-160°W; 30–40°N) could potentially account for the patchy distribution of squid habitat hotspots in May. Moreover, from June to July, an evident east-west gradient in squid habitat hotspots separated at 180°, where the western regions tended to have highly variable squid habitat (Fig 5B and 5C) and hotspots with relatively low HPI (Fig 6B and 6C), could also be potentially attributed to the association of squids to eddy features with geographic positions changing from year to year. Despite the presence of both warm- and cold-core eddies in the study area, squid habitat hotspots were also associated with the former as evident from higher number of warm-core eddies within and around the periphery of squid habitat hotspots (Fig 8A–8C). Warm-core eddies exhibit convergent flow and could aggregate prey species and create favorable foraging conditions for apex predators including squids [63, 64]. East of the dateline (180°), however, the spatial expansion of the squid potential habitat hotspots is likely from the association of squids to the seasonally-migrating, albeit quasi-permanent TZCF and consequent utilization of the productive waters of the subarctic (Fig 7A–7C). The TZCF is a sharp surface chlorophyll-*a* gradient that delineates the less productive subtropical waters with more productive subarctic waters in the north [65]. As the season transitioned from late spring (May) to mid-summer (July), squid habitat hotspots sit atop of and move closer to TZCF, presumably providing good foraging opportunities for neon flying squid (Fig 7A–7C). Polovina et al. [25] suggested that top pelagic predators track the TZCF during their northward feeding migration as this frontal feature may coincide with a convergent front along which, prey species for marine predators are accumulated. In this study, squids are also likely to track the position of the TZCF to optimize their feeding conditions in the central North Pacific by acting as a biophysical cue to maintain their foraging habitat well within a high productivity zone (Fig 7A–7C) throughout the summer season. This possibly accounted for the wider regions of predicted squid habitat hotspots west of the dateline from June and July.

In all the monthly models, squid habitat hotspots were largely influenced by SST such that the northern boundary of these regions are defined by temperature higher than 10°C (Fig 4A–4C). This result is in agreement with the findings of earlier studies reporting that distribution of neon flying squid occurs at zones above 10°C [17, 66]. The second most important variable influencing squid habitat was SSH and could be possibly related to the energetic mesoscale activity, as the central North Pacific appears to be an eddy-rich zone (Fig 8A–8C). While all the monthly habitat models highlighted the importance of SST and SSH to squid potential habitat, other environmental factors showed different amounts of model contribution from May to July, presumably reflecting differences in the physical oceanographic features associated with squid habitat. In May, for instance, deeper MLDs (Fig 4A, right panel) were found favorable to squid habitat in the nutrient-limited KOTZ, as this condition provide high nutrient flux to the surface, thereby supporting primary productivity [67]. In June, however, moderate to strong eastward geostrophic flow (Fig 4B, right panel) appeared favorable to the formation of squid potential habitat and possibly important for the accumulation of passive prey species through advective processes from the west to the central North Pacific [68, 69], where higher primary productivity rates and phytoplankton concentration are generally observed from late spring to early summer [70]. In July, as the squid potential habitat shifted further north towards the subarctic frontal zone (SAFZ), SSS (Table 2) showed a model variable contribution comparable to that of SSH. The SAFZ is generally characterized by presence of strong thermohaline fronts [71, 72] which could possibly explain the increase in the model variable contribution of SSS during this period. These findings suggest that the relative model contribution of environmental parameters differ across space and time and these variations in turn, could further affect the dynamics of potential squid habitat hotspots.

## Summary and Conclusion

The squid potential habitat hotspots in the central North Pacific were influenced by spatial and temporal oceanographic features during the seasonal transition from late spring to summer. The distribution patterns of the habitat hotspots from May to July also highlighted 2 distinct zonal and meridional gradients separated at 180° and 40°N, respectively: (1) 160°E-180°; 37–40°N and (2) 180°-160°W; 40–44°N. Region 1 corresponded to the highly variable habitat with lower persistence index relative to region 2 and their spatio-temporal characteristics are presumably driven by the oceanographic features to which, squids are likely to associate with during their feeding migration. The potential relevance of the oceanic eddies and TZCF to the dynamics of squid habitat hotspots in central North Pacific reinforces the fundamental idea that the physical processes leading to the formation and persistence of pelagic hotspots vary and operate through different spatial and temporal scales [5].

## Supporting Information

S1 Fig Frequency histogram and summary statistics of the squid catch per unit effort (CPUE) for each occurrence point used for habitat model construction from May to July 1999–2010. (PDF)
