# Supplementary material for: Identifying Pelagic Habitat Hotspots of Neon Flying Squid in the Temperate Waters of the Central North Pacific
Source: PLoS One. 2015 Nov 16;10(11):e0142885. doi: 10.1371/journal.pone.0142885 (PMC4646643; doi:10.1371/journal.pone.0142885)
Supplement: S1 Fig — (PDF) [file pone.0142885.s001.pdf]

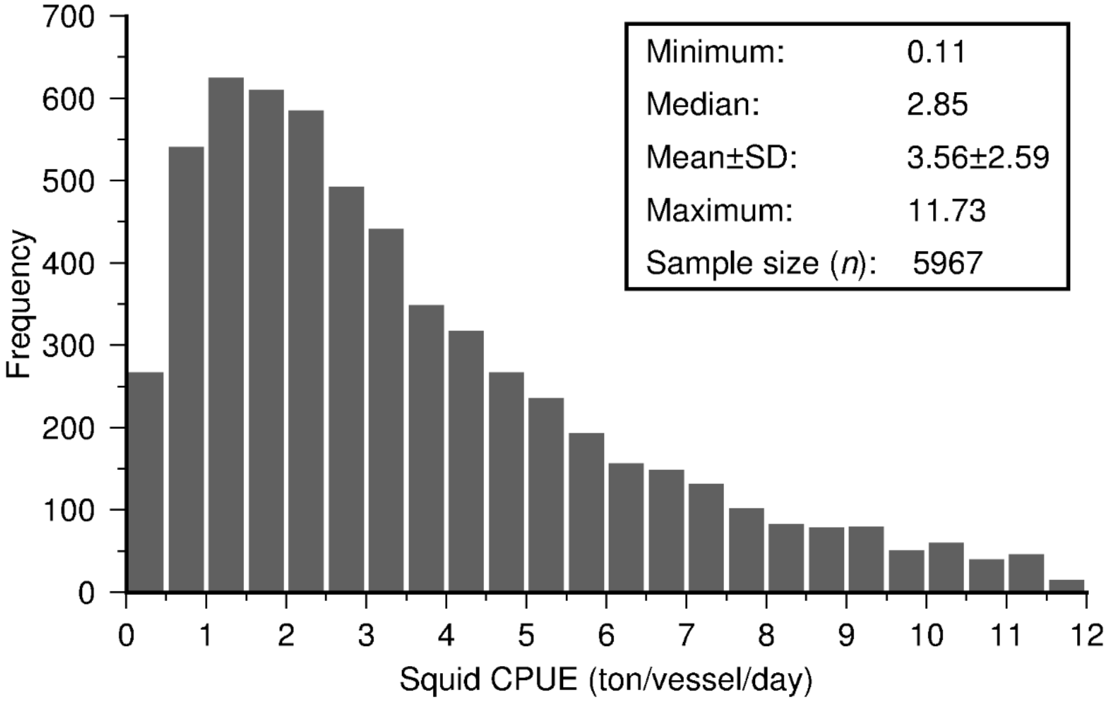

S1 Fig. Frequency histogram and summary statistics of the squid catch per unit effort (CPUE) for each occurrence point used for habitat model construction from May-July 1999-2010.
